# Lead (Pb) exposure from outdoor air pollution: a potential risk factor for cervical intraepithelial neoplasia related to HPV genotypes

**DOI:** 10.1007/s11356-021-17608-x

**Published:** 2021-12-13

**Authors:** Ji Zhang, Seyed Ali Nazeri, Amir Sohrabi

**Affiliations:** 1 Department of Medical Epidemiology and Biostatistics, Karolinska Institutet, Nobels väg 12A, PO Box 171 65, Stockholm, Sweden; 2 Research Center of Health Reference Laboratory, Ministry of Health and Medical Education, Tehran, Iran

**Keywords:** Pb (lead), HPV, Infection, Cervical cancer, Air pollution, Iran

## Abstract

Human papillomavirus genotypes (HPVs) have been confirmed to be the major cause of cervical intraepithelial neoplasia (CIN) that remains to be one of the most common women cancers around the world. It seems other risk factors have synergistic effects on cervical cancer occurrence including smoking, dietary pattern, sexual behavior, ethnicity, epigenetics, and environmental hazardous materials. Our study characterized the potential cancerous role of lead (Pb) as a common toxic environmental pollutant agent on CIN outcomes. Lead concentration was quantified using an atomic absorption spectrometer in liquid-based cytology specimens of 40 CIN-HPV positive subjects, 50 HPV infected non-cancerous cases, and 43 non-HPV infected/non-cancerous women. Pb concentration was 5.5 (4.7–6.4) μg/dL, 4.7 (4.2–8.7) μg/dL, and 4.7 (4.5–5.4) μg/dL in the CIN-HPV positive group, HPV infected non-cancerous cases, and non-HPV infected/non-cancerous group, respectively. The results showed higher Pb concentration is associated with higher risk for cervical malignancy in comparison with non-HPV infected/non-cancerous subjects, after controlling for age effect (aOR = 4.55, 95% CI: 1.55–15.07, *P* < 0.01). Our finding suggested a direct significant association between Pb accumulation and CIN existence. The consequences need to be further validated by including more relevant risk factors and controlling the confounders for better understating of Pb impact from outdoor air pollution on cervical cancer progression.

## Introduction

Tehran, a metropolitan city with a population of more than 10 million people, is one the most polluted areas throughout the world. Air pollution is a life-threating factor caused by urbanization and industrialization especially due to fuel vehicles. Therefore, it seems traffic congestion is a common source of Pb emission to the environment (Ali Asghar 2021; Kermani et al. 2016; Khorrami et al. 2021).

Cervical cancer is the fourth most common diagnosed cancer among women and causes substantial deaths globally. Although cervical cancer incidence has been declining in recent years due to effective screening and vaccination programs in developed countries. On the other hand, cervical cancer related to human papillomavirus (HPV) remains to be a severe public health concern in undeveloped countries lacking preventive medical resources and organized health screening programs. The incidence of cervical cancer varies in undeveloped and developed communities from 11.3 to 18.8 per 100,000 women in 2020, respectively. Several potential risk factors including HPV infection, life style, and self-awareness have been associated to cervical and genital malignancies (Momenimovahed and Salehiniya 2018; Sohrabi and Hajia 2017; Sung et al. 2021; Vafaeinezhad et al. 2018). Persistent and colonization of high-risk HPV genotypes in the genital tract can lead to cervical neoplasia (Hajia and Sohrabi 2018; Stanley 2010). In addition, multiple environmental factors are also shown to be involved in risk profile of cervical cancer. Not surprisingly, factors associated with acquisition or pathogenic progress of HPV play an important role for precancerous abnormalities to cervical cancer. Those factors include early age of first intercourse, sexually transmitted infections (STIs), multiple sexual partners, multiple pregnancies, tobacco smoking, and lack of fruits and vegetables in diets (Cohen et al. 2019; Sohrabi et al. 2017). Besides, accumulating evidence is suggesting a contributing risk effect of heavy metals as well. Heavy metals refer to metallic elements with relative higher density of greater than 5 g/cm^3^. Some of them are essential for biological progress, while they can be deleterious and may cause cancer after exposure to high quantity or long-term exposure to even small quantity (Engwa et al. 2019). Among a wide range of heavy metals in the environment, lead (Pb) is a kind of widely distributed toxic environmental pollutant in the universe. Pb is classified as possible carcinogenic group 2B to humans (Fasinu and Orisakwe 2013; IARC 2006). It can be accumulated and identified from soil waste, drinking water, smoke, air, fruits and vegetables due to air pollution by industrial factories and fuel vehicles (traffic congestion). Scientific literatures have shown a link between Pb and gastrointestinal, lung, bladder, and head-neck cancers (Cobanoglu et al. 2010; Khlifi and Hamza-Chaffai 2010; Sadetzki et al. 2000; Turkdogan et al. 2003; Yuan et al. 2016).

It would seem findings about the association between Pb concentration and cervical cancer are limited. The current study mainly focuses on characterizations of Pb concentration on women cervical scrapping specimens who live in Tehran, which is one of the most polluted cities in the world. Hence, we have appraised and assessed the association between Pb concentration in women who suffered from CINs related to HPV genotypes in comparison with non-HPV infected/non-cancerous outcomes.

## Materials and methods

### Study population and data collection

In order to meet ethical considerations, the study was performed in accordance with the 1964 Declaration of Helsinki and its later amendments. Each cancer subject was informed about the objectives of the study and has signed a consent form before entering the study. The controls were residual of archival specimens from other studies. The necessary clinical data was collected from medical documents. The details of participants’ clinical information are not mentioned in the publication. A total of 133 liquid-based cytology specimens were obtained from studies that described and published previously (Moharreri and Sohrabi 2021; Sohrabi et al. 2014; Sohrabi et al. 2016). The samples were included 40 cases with cervical intraepithelial neoplasia (CIN-HPV positive group), 50 HPV infected non-cancerous cases, and 43 non-HPV infected/non-cancerous women. Women specimens were also confirmed by histopathological examinations to identify any pathological changes. All population studies were tested for HPV genotyping by approved HPV detection kits (Moharreri and Sohrabi 2021). The HPV genotyping consequences were divided into different categories such as high-risk HPVs 16 and 18, low-risk HPVs 6 and 11, single and multiple high-risk HPV genotypes for statistical analysis.

### Lead (Pb) measurement

Pb was measured using an atomic absorption spectrometer (Agilent technologies/200 Series^©^, USA). The AA spectrometer was equipped with a GTA120 (graphite tube atomizer) and an auto sampler. Pyrolytic ally–coated furnace tubes were employed and trace metal–free polycarbonate tubes were used for sample preparation. GFAAS conditions: 283.3-nm wavelength, 7A slit, D2 background correction, grooved furnace tube. Dry: ambient to 125 °C in 15-s ramp, 5-s hold. Ash: 125 °C to 600 °C in 45-s ramp, 20-s hold. Atomize: 600 °C to 2400 °C in fast ramp or step, 5-s hold.

The Milli-Q water was purified by de-ionization with a Milli-Q system (Millipore) for washing all laboratory wares, solutions, and standards preparation. In addition, all reagents were obtained from Merck Co. Working standards were prepared daily by serial dilution of a master standard with 0.1% v/v nitric acid. Dilution ranges of Pb standards were made and vortexed by 0, 1, 5, 10, 25, 50, and 100 μg/dL for working standard solutions. Calibration was also performed directly using aqueous standards.

One hundred microliters of samples was diluted with 400 μL of “Matrix modifier” (containing 0.1% v/v nitric acid, 0.2% m/v ammonium dihydrogen phosphate, and 0.5% m/v Triton X-100). The Pb concentration of samples was calculated from integrated absorbance measurements and calibration graph. Analysis was performed with 20-μL loads under the following GFAAS conditions.

The method was also evaluated and verified for accuracy and precision measurements. The limit of detection was defined as 3 times of blank standard deviation (SD) signal that was 0.2 μg/dL, corresponding to a limit of quantification (10× SD) of 0.6 μg/dL. Precision was 6.7% at 10 μg/dL (*n =* 20) and 2.7 at 25 μg/dL (*n* = 20). The recovery of 25 μg/dL was 97.2%. The accuracy was checked by analyzing standard reference materials: Seronorm™ (Trace Elements Serum), an accuracy control for analysis of trace elements and heavy metals (Md Noh et al. 1977; Taupeau et al. 2001; World Health Organization 2011).

### Statistical analysis

Pb concentration was investigated as numerical variable and presented in the CIN-HPV positive group, HPV infected non-cancerous cases, and non-HPV infected/non-cancerous subjects. As the Pb concentration had a skewed distribution in the study population, it was summarized using median value and corresponding interquartile range in each group and compared using non-parametric methods. Specifically, we used the Mann-Whitney *U* test to compare the difference of Pb concentration between case and control groups. The Kruskal-Wallis test was also used to compare the difference between CIN grades. Then, we divided the age into greater than or equal to 35 and below 35 years old, and Pb concentration between the two age groups was compared in each population study. Furthermore, the Spearman correlation analysis was performed to estimate correlation between Pb concentration and subject’s age in each group. We further divided Pb exposure into low concentration and high concentration by its median value among all subjects and categorized it into low, middle, and high level groups by its quartile concentrations. The association was investigated between different levels of Pb exposure and CIN using the logistic regression model. Controlling was done for age effect and HPV genotypes. CIN cases were compared with HPV positive and HPV negative controls, separately. Results were reported as odds ratios (ORs) and corresponding 95% confidence intervals (CIs).

In addition, we studied the Pb concentration in different HPV groups based on their genotypes. Findings were presented as boxplots and summarized using median values and interquartile ranges. The difference of Pb concentrations was compared between HPV infected and non-HPV infected women by the Mann-Whitney *U* test. Comparison of Pb concentration was also performed between multiple HPV genotypes using the Kruskal-Wallis test. The statistical analysis was conducted using R software (version 3.6.1), and a two-tailed *P* value ≤ 0.05 was regarded to be statistically significant.

## Results

### Pb consequences

Pb concentration was measured on 133 women and median concentration of Pb was higher in the cancer group compared with the total control group (*P* < 0.01). As a result, when controls were divided into HPV infected and non-HPV infected, Pb level in the case group was only statistically higher than non-HPV infected women (*P* < 0.01). The median Pb concentration was also different within CIN grades-HPV positive group (*P* = 0.019). The outcomes are presented in Table 1.

**Table 1:** Characteristics of lead (Pb) concentration in CIN subjects and controls (*n* = 133)

Research group	Pb concentration (μg/dL)	
	Median	Interquartile range
CIN (*N* = 40)	5.5	4.7–6.4
CIN I (*N* = 6)	6.6	6.4–7.0
CIN II (*N* = 6)	5.0	4.7–5.8
CIN III (*N* = 28)	5.4	4.7–6.2
Control (*N* = 93)	4.7	4.3–5.8
Non-cancerous/HPV infected (*N* = 50)	4.7	4.2–8.7
Non-cancerous/non-HPV infected (*N* = 43)	4.7	4.5–5.4
**Comparisons**	***P*** **value**	
CIN versus overall controls	< 0.01^a^	
CIN versus HPV infected control	0.080^a^	
CIN versus non-HPV infected control	< 0.01^a^	
Comparison between CIN grades	0.019^b^	

Abbreviations: *CIN*, cervical intraepithelial neoplasia; *HPV*, human papillomavirus; *Pb*, lead

^a^The *P* value comparison between two indicated groups by the Mann-Whitney *U* test

^b^The *P* value comparison between three groups by the Kruskal-Wallis test

### Pb concentration and age

The difference of Pb concentrations was also examined in two age groups and its potential linear correlation with age as a numerical variable, in each study group. Outcomes are shown in Table 2. In the total control group, Pb level was higher in the age below 35 years old, compared to the age greater than 35 years old but without linear correlation (*P*_difference_ = 0.04). In the non-HPV infected/non-cancerous group, Pb concentration was higher in the younger age group and was negative linearly correlated with age (*P*_difference_ = 0.01; *r* = −0.29, *P*_correlation_ < 0.01), while such a pattern was not observed in HPV infected non-cancerous cases.

**Table 2:** Pb concentrations in different age groups within population study and its linear correlation

Population study	Pb concentration		*P* ^a^_difference_	*r*	*P* ^b^_correlation_
	Median	Interquartile range			
CINs					
< 35 (*N* = 6)	5.1	4.7–6.4	0.58	0.00	0.972
≥ 35 (*N* = 34)	5.6	4.7–6.4			
Controls					
< 35 (*N* = 60)	5.0	4.5–6.3	0.04	−0.13	0.072
≥ 35 (*N* = 33)	4.6	4.3–4.8			
Non-cancerous/HPV infected					
< 35 (*N* = 36)	4.8	4.2–10.1	0.69	−0.01	0.927
≥ 35 (*N* = 14)	4.6	4.3–4.9			
Non-cancerous/non-HPV infected					
< 35 (*N* = 24)	5.1	4.6–5.8	0.01	−0.29	< 0.01
≥ 35 (*N* = 19)	4.6	4.3–4.7			

Abbreviations: *CIN*, cervical intraepithelial neoplasia; *HPV*, human papillomavirus; *Pb*, lead

^a^The difference comparison of Pb concentration between two age groups, Mann-Whitney *U* test

^b^The *P* value for the Spearman correlation between Pb concentration and age

### Association between Pb level and CINs

The association between binary Pb level and CIN was compared between the CIN-HPV positive group, HPV infected-non cancerous subjects, and the non-HPV infected/non-cancerous control group. The results are shown in Table 3, separately. Pb exposure of women studied was dichotomized by the median concentration (4.8 μg/dL). After controlling for age, higher Pb level was associated with a higher risk of CIN in comparison with the total control group (aOR = 3.61, 95% CI: 1.43–9.87, *P* < 0.01), as well as in comparison with the control group without HPV infection (aOR = 4.55, 95% CI: 1.55–15.07, *P* < 0.01). No statistical significance was observed between Pb and CIN-HPV positive group in comparison with the HPV infected control group. The association between ternary Pb level and CIN (low: < 4.6, middle: 4.6–5.7, high: > 5.7 μg/dL) was conducted in comparison with different control groups. After controlling for age effect in the regression model, higher level of Pb concentration was associated with a higher risk for CIN in comparison with the total control group (aOR = 5.72, 95% CI: 1.87–19.73, *P* < 0.01). The detailed outcomes are mentioned in Table 4. Moreover, greater OR was observed when compared with the non-HPV infected/non-cancerous control group (aOR = 7.01, 95% CI: 1.89–29.91, *P* < 0.01).

**Table 3:** Risk analysis for CIN in association with higher level of Pb concentration, which is categorized by its median level in all subjects, comparing with different control groups

	*N* (case/control)	OR^a^ (95% CI)	*P*	aOR^b^ (95% CI)	*P*
Compare with overall controls					
Low (< 4.8)	11/50	1.00 (Ref.)	< 0.01	1.00 (Ref.)	< 0.01
High (≥ 4.8)	29/43	3.07 (1.40–7.09)		3.61 (1.43–9.87)	
Compare with non-cancerous/HPV infected					
Low (< 4.8)	11/26	1.00 (Ref.)	0.021	1.00 (Ref.)	0.104
High (≥ 4.8)	29/24	2.86 (1.20–7.14)		2.43 (0.85–7.36)	
Compare with non-cancerous/non-HPV infected					
Low (< 4.8)	11/24	1.00 (Ref.)	0.010	1.00 (Ref.)	< 0.01
High (≥ 4.8)	29/19	3.33 (1.35–8.59)		4.55 (1.55–15.07)	

Abbreviations: *CIN*, cervical intraepithelial neoplasia; *HPV*, human papillomavirus; *Pb*, lead; *OR*, odds ratio; *aOR*, adjusted odds ratio; *95% CI*, 95% confidence interval

^a^Unadjusted OR estimated by the logistic regression model

^b^Adjusted OR estimated by the logistic regression model, controlling for women’s age effect in the model

**Table 4:** Risk analysis for CIN in association with higher level of Pb concentration, which is categorized by its tertile value in all subjects, comparing with different control groups (*n* = 133)

	*N* (case/control)	OR^a^ (95% CI)	*P*	aOR^b^ (95% CI)	*P*
Compare with overall controls					
Low (< 4.6)	8/41	1.00 (Ref.)		1.00 (Ref.)	
Middle (4.6–5.7)	13/27	2.47 (0.92–7.00)	0.078	2.34 (0.72–8.07)	0.162
High (> 5.7)	19/25	3.90 (1.53–10.70)	< 0.01	5.72 (1.87–19.73)	< 0.01
Compare with non-cancerous/HPV infected					
Low (< 4.6)	8/22	1.00 (Ref.)		1.00 (Ref.)	
Middle (4.6–5.7)	13/12	2.98 (0.98–9.55)	0.058	2.26 (0.57–9.43)	0.250
High (> 5.7)	19/16	3.27 (1.12–9.70)	0.027	3.40 (0.99–12.94)	0.059
Compare with non-cancerous/non-HPV infected					
Low (< 4.6)	8/19	1.00 (Ref.)		1.00 (Ref.)	
Middle (4.6–5.7)	13/15	2.06 (0.69–6.46)	0.203	2.01 (0.54–7.95)	0.305
High (> 5.7)	19/9	5.01 (1.65–16.58)	< 0.01	7.01 (1.89–29.91)	< 0.01

Abbreviations: *CIN*, cervical intraepithelial neoplasia; *HPV*, human papillomavirus; *Pb*, lead; *OR*, odds ratio; *aOR*, adjusted odds ratio; *95% CI*, 95% confidence interval

^a^Unadjusted OR estimated by the logistic regression model

^b^Adjusted OR estimated by the logistic regression model, controlling for women’s age effect

### Pb concentration and HPV genotypes

**Fig. 1:**
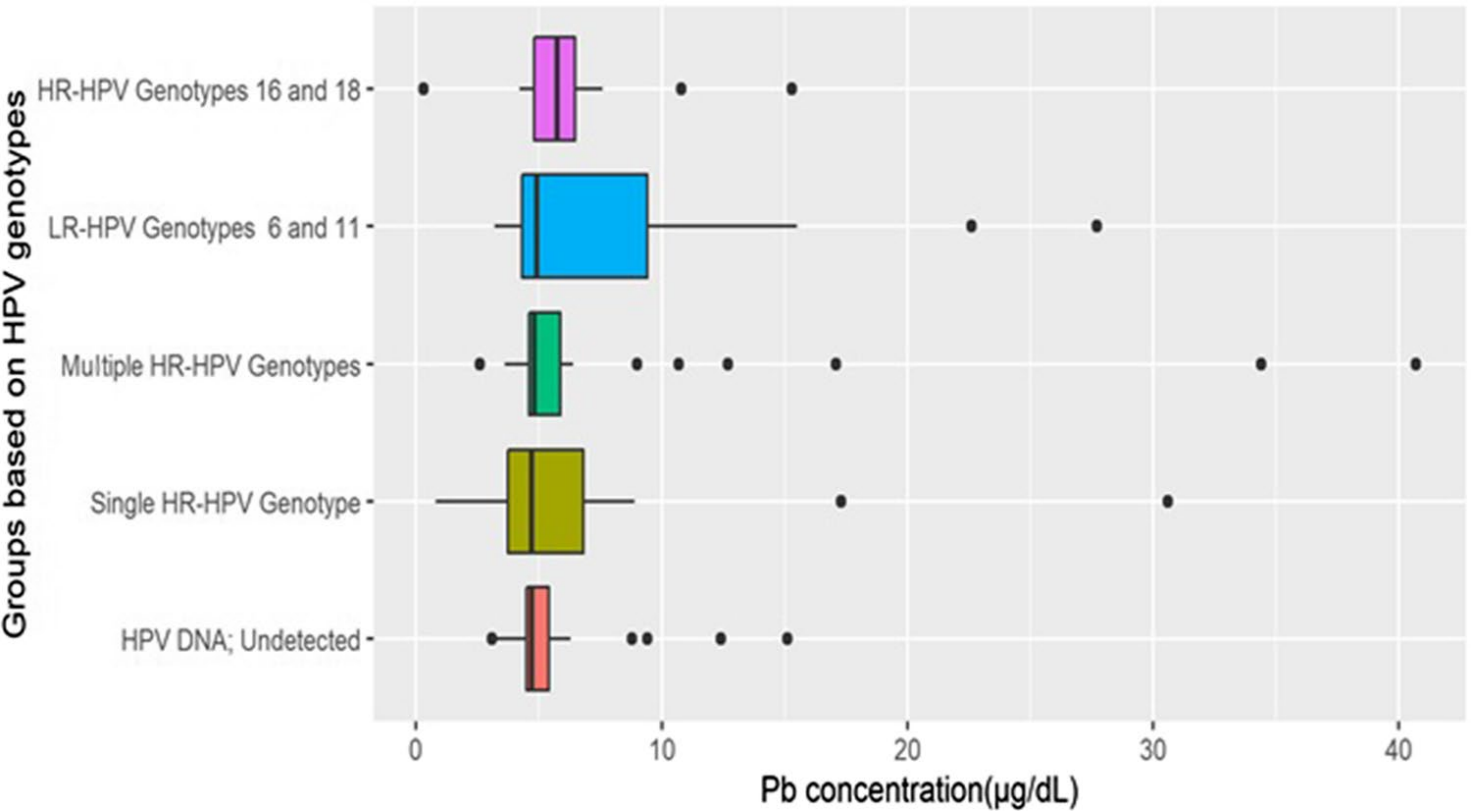
Boxplots of Pb concentration in each group based on HPV genotypes. In the boxplot of each group, the median concentration is vertical bold line inside the box, and the 25th and 75th percentiles are left and right vertical bounds of the box, respectively. The horizontal line represented the range between 1.5 times interquartile below 25th percentile and 1.5 times interquartile above 75th percentile. In addition, the points were measurements of below or above 1.5 time’s interquartile range

Pb concentrations of different HPV genotype groups are demonstrated in Fig. 1. Moreover, the difference of Pb concentration was compared between women with and without HPV infection among all subjects or restricted in the control group. It was also compared within groups with different HPV genotypes. Pb concentrations were not differently distributed in different HPV genotype groups. The detailed results are not shown. HPV genotype was also included as a confounding variable in the model comparing cases with HPV infected control to assess the association between Pb level and outcome, although no statistically significant difference was observed (Table 5).

**Table 5:** Women risk analysis for CINs comparing to HPV infected controls (controlling for HPV genotype as a confounder)

	*N* (case/control)	aOR (95% CI)^a^	*P*
Pb concentration categorized by its median value			
Low (< 4.8)	11/26	1.00 (Ref.)	
High (≥ 4.8)	29/24	1.33 (0.35–5.03)	0.673
Pb concentration categorized by its tertile values			
Low (< 4.6)	8/22	1.00 (Ref.)	
Middle (4.6–5.7)	13/12	2.04 (0.40–10.95)	0.392
High (> 5.7)	19/16	3.13 (0.61–18.38)	0.180

Abbreviations: *CIN*, cervical intraepithelial neoplasia; *HPV*, human papillomavirus; *Pb*, lead; *aOR*, adjusted odds ratio; *95% CI*, 95% confidence interval

^a^Adjusted OR estimated by the logistic regression model, controlling for age and HPV genotypes

## Discussion

Women could possibly be more exposed to Pb, as a toxic element for multiple organs and cancers at even a low dose after a long-term exposure, through ambient environment, cooking, and cosmetics. In line with our results, a previous study also reported a higher Pb level in CIN endo-cervical tissues, compared to histological normal tissues. Notably, their study had 3 CIN cases, while our study focused on a bigger sample size, ensuring a better statistical power (Rzymski et al. 2016; Sanders et al. 2015). There was not any clear significant association between Pb and cervical cancer. In addition, the scientific literatures are limited in genital carcinoma issues related to Pb. Many scientific efforts confirmed there is a link between Pb exposure and human malignancies. DNA and chromosomal damages, Pb accumulation in organs, and tumorigenesis through impairment of DNA repair system and change of miRNA expression in cervix (through epigenetic regulations and immunotoxin effects by dysregulating cytokine productions, promoting inflammation and expression altering of T-helper cell activity) are side effects of lead. Therefore, finding any association and synergistic effects between Pb and carcinogen heavy metals e.g. Cadmium related to cervical cancer should be considered in future studies (Caffo et al. 2014; Fenga et al. 2017; Kumar 2018; Marouf 2018; Matovic et al. 2015; Rzymski et al. 2016; Sanders et al. 2015; Silbergeld 2003; Silbergeld et al. 2000; Wilk et al. 2017).

Specifically, it seems women smokers have a higher Pb concentration in the endo-cervical tissues than non-women smokers, although we could not access smoking habit data of the current study. However, it seems the prevalence rate of tobacco smoking is less than 5% among Iranian women (Sohrabi et al. 2020).

Microbial pathogens might also play a synergistic role for carcinogenic heavy metals and trace element existence in cervical cancer progression (Fenga et al. 2017; Rzymski et al. 2016). Nevertheless, none of proposed mechanisms could fully explain of Pb carcinogenic role or its differential expressions in multiple organs. We focused to find any association between HPV genotypes and Pb that there was no significant difference. It seems further studies are still needed to better understand and clarify Pb effect on human health.

Our study adds up to the direct evidence that higher Pb level accumulated in tissue of cervical neoplasia. It seems it is associated with higher risk of cancerous changes. There are also significant drawbacks in our study. Firstly, our study could not exclude the effects of factors that may have a significant impact on Pb exposure such as smoking habits, education level, living conditions, and working environment. Albeit, smoking habit is not common in Iranian women. We considered the various risk factors and variables in the study questionnaire, but they have missed meanwhile the sample collection. Therefore, insufficient factors were excluded for biostatistical analysis. Secondly, we could not elucidate the possible causal relationship between Pb exposure and cervical cancer in the current setting. Pb could trigger cancerous changes in cervical tissues related to HPV genotypes.

## Conclusion

Here, the study showed an association between increase of cervical Pb concentration and progression of cervical intraepithelial neoplasia in comparison with non-HPV infected/non-cancerous subjects, after controlling for age effect. As a result, the higher level of cervical tissue Pb concentration was associated with a higher risk for CIN subjects in comparison with total control studied and non-HPV infected control. Therefore, further dedicated attempts are needed to validate the outcomes by including more relevant risk factors, appraising the source of Pb exposures and finding any correlations particularly from outdoor air pollution and its consequences on cervical cancer progression.

## Data Availability

All data are mentioned in the body of manuscript, tables, and figure.

## References

[CR1] Ali Asghar P (2021). Spatial-geographical analysis of urbanization in iran. Humanities Social Sci Commun.

[CR2] Caffo M, Caruso G, Fata GL, Barresi V, Visalli M, Venza M (2014). Heavy metals and epigenetic alterations in brain tumors. Curr Genomics.

[CR3] Cobanoglu U, Demir H, Sayir F, Duran M, Mergan D (2010). Some mineral, trace element and heavy metal concentrations in lung cancer. Asian Pac J Cancer Prev.

[CR4] Cohen PA, Jhingran A, Oaknin A, Denny L (2019). Cervical cancer. Lancet.

[CR5] Engwa GA, Ferdinand PU, Nwalo FN, Unachukwu MN (2019) Mechanism and health effects of heavy metal toxicity in humans, Poisoning in the modern world - new tricks for an old dog 10. IntechOpen. 10.5772/intechopen.82511

[CR6] Fasinu P, Orisakwe OE (2013). Heavy metal pollution in sub-saharan Africa and possible implications in cancer epidemiology. Asian Pac J Cancer Prev.

[CR7] Fenga C, Gangemi S, Di Salvatore V, Falzone L, Libra M (2017). Immunological effects of occupational exposure to lead (review). Mol Med Rep.

[CR8] Hajia M, Sohrabi A (2018). Possible synergistic interactions among multiple HPV genotypes in women suffering from genital neoplasia. Asian Pac J Cancer Prev.

[CR9] IARC (2006) Working Group on the Evaluation of Carcinogenic Risks to Humans Inorganic and organic lead compounds. IARC monographs on the evaluation of carcinogenic risks to humans, 87, 1. 87:1-471 PMC4781642 17191367

[CR10] Kermani M, Dowlati M, Jonidi Jafari A, Rezaei Kalantary R (2016). A study on the comparative investigation of Air Quality Health Index (AQHI) and its application in tehran as a megacity since 2007 to 2014. J Res Environ Health.

[CR11] Khlifi R, Hamza-Chaffai A (2010). Head and neck cancer due to heavy metal exposure via tobacco smoking and professional exposure: a review. Toxicol Appl Pharmacol.

[CR12] Khorrami Z, Pourkhosravani M, Rezapour M, Etemad K, Taghavi-Shahri SM, Kunzli N (2021). Multiple air pollutant exposure and lung cancer in Tehran, Iran. Sci Rep.

[CR13] Kumar S (2018). Occupational and environmental exposure to lead and reproductive health impairment: an overview. Indian J Occup Environ Med.

[CR14] Marouf BH (2018). Association between serum heavy metals level and cancer incidence in Darbandikhan and Kalar area, Kurdistan region, Iraq. Niger J Clin Pract.

[CR15] Matovic V, Buha A, Ethukic-Cosic D, Bulat Z (2015). Insight into the oxidative stress induced by lead and/or cadmium in blood, liver and kidneys. Food Chem Toxicol.

[CR16] Md Noh MF, Ismail Z, Surif A (1977). A rapid measurement of lead in whole blood by graphite furnace atomic absorption spectrometer. Malaysian J Biochem Mol Biol.

[CR17] Moharreri M, Sohrabi A (2021). Characteristics of hsv-2, m. Genitalium and c. Trachomatis in HPV genotypes associated with cervical intraepithelial neoplasia and genital infections. Infect Disord Drug Targets.

[CR18] Momenimovahed Z, Salehiniya H (2018). Cervical cancer in Iran: integrative insights of epidemiological analysis. Biomedicine (Taipei).

[CR19] Rzymski P, Niedzielski P, Rzymski P, Tomczyk K, Kozak L, Poniedzialek B (2016). Metal accumulation in the human uterus varies by pathology and smoking status. Fertil Steril.

[CR20] Sadetzki S, Bensal D, Blumstein T, Novikov I, Modan B (2000). Selected risk factors for transitional cell bladder cancer. Med Oncol.

[CR21] Sanders AP, Burris HH, Just AC, Motta V, Amarasiriwardena C, Svensson K (2015). Altered miRNA expression in the cervix during pregnancy associated with lead and mercury exposure. Epigenomics.

[CR22] Silbergeld EK, Waalkes M, Rice JM (2000). Lead as a carcinogen: experimental evidence and mechanisms of action. Am J Ind Med.

[CR23] Silbergeld EK (2003). Facilitative mechanisms of lead as a carcinogen. Mutat Res.

[CR24] Sohrabi A, Mirab-Samiee S, Modarresi MH, Izadimood N, Azadmanesh K, Rahnamaye-Farzami M (2014). Development of in-house multiplex real time PCR for human papillomavirus genotyping in iranian women with cervical cancer and cervical intraepithelial neoplasia. Asian Pac J Cancer Prev.

[CR25] Sohrabi A, Rahnamaye-Farzami M, Mirab-Samiee S, Mahdavi S, Babaei M (2016). Comparison of in-house multiplex real time PCR, diagcor genoflow HPV array test and INNO-LiPA HPV genotyping extra assays with LCD- array kit for human papillomavirus genotyping in cervical liquid based cytology specimens and genital lesions in Tehran, Iran. Clin Lab.

[CR26] Sohrabi A, Hajia M (2017). Cervical cancer and genital infections: assessment of performance and validation in human papillomavirus genotyping assays in Iran, its neighbouring countries and Persian Gulf area. Iran J Pathol.

[CR27] Sohrabi A, Hajia M, Jamali F, Kharazi F (2017). Is incidence of multiple HPV genotypes rising in genital infections?. J Infect Public Health.

[CR28] Sohrabi MR, Abbasi-Kangevari M, Kolahi AA (2020) Current tobacco smoking prevalence among Iranian population: a closer look at the STEPS surveys. Front Public Health 16:571062. 10.3389/fpubh.2020.571062 10.3389/fpubh.2020.571062 PMC7784444 33415092

[CR29] Stanley M (2010). Pathology and epidemiology of HPV infection in females. Gynecol Oncol.

[CR30] Sung H, Ferlay J, Siegel RL, Laversanne M, Soerjomataram I, Jemal A (2021). Global Cancer Statistics 2020: GLOBOCAN estimates of incidence and mortality worldwide for 36 cancers in 185 countries. CA Cancer J Clin.

[CR31] Taupeau C, Poupon J, Nome F, Lefevre B (2001). Lead accumulation in the mouse ovary after treatment-induced follicular atresia. Reprod Toxicol.

[CR32] Turkdogan MK, Kilicel F, Kara K, Tuncer I, Uygan I (2003). Heavy metals in soil, vegetables and fruits in the endemic upper gastrointestinal cancer region of Turkey. Environ Toxicol Pharmacol.

[CR33] Vafaeinezhad Z, Kazemi Z, Mirmoeini M, Piroti H, Sadeghian E, Mohammad Ali-Vajari M (2018). Trends in cervical cancer incidence in Iran according to national cancer registry. J Mazandaran Univ Medical Sci.

[CR34] Wilk A, Kalisinska E, Kosik-Bogacka DI, Romanowski M, Rozanski J, Ciechanowski K (2017). Cadmium, lead and mercury concentrations in pathologically altered human kidneys. Environ Geochem Health.

[CR35] World Health Organization (2011) Brief guide to analytical methods for measuring lead in blood. In Brief guide to analytical methods for measuring lead in blood

[CR36] Yuan W, Yang N, Li X (2016). Advances in understanding how heavy metal pollution triggers gastric cancer. Biomed Res Int.

